# Environmental, psychological, and social influences on physical activity among Japanese adults: structural equation modeling analysis

**DOI:** 10.1186/1479-5868-7-61

**Published:** 2010-08-05

**Authors:** Kaori Ishii, Ai Shibata, Koichiro Oka

**Affiliations:** 1 Faculty of Sport Sciences, Waseda University, Saitama, Japan

## Abstract

**Background:**

An understanding of the contributing factors to be considered when examining how individuals engage in physical activity is important for promoting population-based physical activity. The environment influences long-term effects on population-based health behaviors. Personal variables, such as self-efficacy and social support, can act as mediators of the predictive relationship between the environment and physical activity. The present study examines the direct and indirect effects of environmental, psychological, and social factors on walking, moderate-intensity activity excluding walking, and vigorous-intensity activity among Japanese adults.

**Methods:**

The participants included 1,928 Japanese adults aged 20-79 years. Seven sociodemographic attributes (e.g., gender, age, education level, employment status), psychological variables (self-efficacy, pros, and cons), social variables (social support), environmental variables (home fitness equipment, access to facilities, neighborhood safety, aesthetic sensibilities, and frequency of observing others exercising), and the International Physical Activity Questionnaire were assessed via an Internet-based survey. Structural equation modeling was conducted to determine associations between environmental, psychological, and social factors with physical activity.

**Results:**

Environmental factors could be seen to have indirect effects on physical activity through their influence on psychological and social variables such as self-efficacy, pros and cons, and social support. The strongest indirect effects could be observed by examining the consequences of environmental factors on physical activity through cons to self-efficacy. The total effects of environmental factors on physical activity were 0.02 on walking, 0.02 on moderate-intensity activity excluding walking, and 0.05 on vigorous-intensity activity.

**Conclusions:**

The present study indicates that environmental factors had indirect effects on walking, moderate-intensity activity excluding walking and vigorous-intensity activity among Japanese adults, especially through the effects on these factors of self-efficacy, social support, and pros and cons. The findings of the present study imply that intervention strategies to promote more engagement in physical activity for population-based health promotion may be necessary.

## Background

Although physical activity has been associated with a lower risk of some types of cancer, cardiovascular disease, diabetes, and obesity [1], a large proportion of the population remains insufficiently physically active. In Japan, only 31% of men and 28% of women engage in thirty minutes or more of exercise two or more times per week [2]. This low percentage of people engaging in exercise can also be observed in many countries in the world. In the United States, less than half of the adult population engages in the recommended amount of thirty minutes of moderate-intensity physical activity on most days of the week [3,4].

An understanding of the contributing factors which encourage individuals to engage in physical activity is important for promoting population-based physical activity. The ecological perspective suggests that physical activity is influenced by an interaction of demographic, psychological, social, and environmental factors [5]. Research on physical activity has traditionally focused on individual demographics and psychosocial correlates such as self-efficacy and social support. However, recent evidence indicates that the environment has long-term effects on population-based health behavior. A number of previous studies have revealed a direct relationship between neighborhood environmental characteristics (e.g., residential density, access to destinations, aesthetics) and physical activity [6-12].

A few studies have suggested that personal variables, such as self-efficacy and social support, can act as mediators of the predictive relationship between the built-environment and physical activity [13-16]. Previous research has demonstrated that the effects of environmental factors were mediated through psychological and social correlates to physical activity [13-16]. Although numerous studies have reported the direct effects of these correlates on physical activity [17-22], only a few studies have examined both the direct and mediated effects of these correlates on physical activity [13-16,23-26].

Moreover, one previous study [27] indicated that correlations between such factors and physical activity varied with reference to different types of behavior, such as walking, exercise, and transport. To the best of our knowledge, only one study from the United States has described the mediated effects of these factors for specific types of physical activities [13]. Hence, an understanding of the direct and indirect influences of factors on specific types of physical activity behavior is important in finding ways to design effective strategies for the promotion of physical activity. To that end, the present study examined how the direct and indirect effects of environmental, psychological, and social factors influenced walking, moderate-intensity activity excluding walking, and vigorous-intensity activity among Japanese adults.

## Methods

### Participants and Data Collection

In the present cross-sectional study, the data were collected in 2008 by a Japanese Internet research service organization by using an Internet-based survey of registrants. The research organization collected approximately 1,150,000 voluntarily registered participants and obtained detailed sociodemographic data for each participant. The present study examined 2,000 Japanese adults aged 20-79 years and stratified these adults by gender and age bracket (20-29 years, 30-39 years, 40-49 years, and >50 years). A total of 7,501 potential respondents were randomly selected, and registrants received a website address via an invitation e-mail from the Internet research service organization (response rate = 26.7%). In order to estimate the representativeness of respondents, the adjusted prevalence for age bracket of married and employed individuals was compared with those in the national survey. The prevalence of married participants was 63.0% for males and 72.5% for females, whereas in the Japanese Population Census Survey of 2005, the adjusted prevalence for age bracket was 61.6% and 65.5%, respectively [28]. With regard to employment status, 78.7% of males and 30.0% of females were employed, whereas the national survey [29] found that 78.3% of males and 59.3% of females worked full-time or part-time. However, the participants may have included a relatively higher proportion of not employed females. This indicates that the study participant used was slightly different from the balance of the general population. The Internet-based questionnaire was provided via a link on a website, and was then accessed by the registrants. All participants signed an informed consent form before answering the questionnaire. The present study received prior approval from the Ethics Committee of the Faculty of Sports Sciences, Waseda University, Japan.

### Measures

#### Sociodemographic attributes

Gender, age, education level, employment status, marital status, living conditions, and household income level were assessed in the self-administered questionnaire. Participants chose the most suitable answer from the categories of education level (graduate school, university, two-year university, career college, high school, junior high school), employment status (office worker, independent businessman, professional, public official, student, housewife, part-time worker, not employed), marital status (married, unmarried), living conditions (number of people cohabitating with, living alone), and household income (<3,000,000 yen, <5,000,000 yen, <7,000,000 yen, <10,000,000 yen, ≥10,000,000 yen).

#### Physical activity

The level of physical activity was estimated from the Japanese version of the short form of the International Physical Activity Questionnaire (IPAQ) [30]. This self-administered questionnaire assessed the frequency and duration of walking for all purposes such as work, transport, and recreation, moderate physical activity, vigorous physical activity, and sedentary activity for a usual week. The test-retest reliability (r = .72-.93) and criterion validity (r = .39) of the scale, measured by using an accelerometer, were confirmed in a previous study of the Japanese population [30]. The total number of weekly minutes of walking, moderate, and vigorous physical activity was computed according to the IPAQ scoring manual [31].

#### Psychological variables

The measurement of self-efficacy for exercise [32] consisted of four items rated using a five-point Likert scale ranging from 1 (strongly disagree) to 5 (strongly agree). The scale assessed the confidence of participants engaging in a physical activity when faced with common barriers such as physical fatigue, poor weather conditions, lack of time, and psychological stress. The two-week test-retest reliability (r = .78) and internal consistency (α = .84) were confirmed in a previous study [32].

Perceived positive (pros) and negative (cons) aspects of exercise included a 10-item pros scale and a 10-item cons scale that were rated using a five-point Likert scale ranging from 1 (strongly disagree) to 5 (strongly agree) [33]. Examples of items in the pros scale were "Regular exercise would help me relieve tension" and "It would be easier for me to perform routine physical tasks if I exercised regularly." Examples of items in the cons scale were "Regular exercise would take too much of my time" and "I would have less time for my family and friends if I exercised regularly." The two-week test-retest reliability (pros: r = .80; cons: r = .77) and internal consistency (pros and cons scales: α = .84) of these scales were confirmed in a previous study [33].

#### Social variables

Social support for exercise [34] was measured using a five-point Likert scale, rated from 1 (strongly disagree) to 5 (strongly agree) with the following items: advice/instruction, understanding/sympathy, encouragement/reinforcement, joint implementation, and compliment/appreciation. These items assessed functional, emotional, and informational social support for exercise. The internal consistency (α = .86) and construct validity (goodness-of-fit index; GFI = .98, adjusted goodness-of-fit index; AGFI = .93, comparative fit index; CFI = 1.00, root mean square error of approximation; RMSEA = .07) of this scale were confirmed in a previous study [34].

#### Environmental variables

Participants' perceptions of their neighborhood environment [17] were measured using a five-item measure including "I possess home fitness equipment (e.g., shoes, pedometer, dumbbells)," "My neighborhood provides facilities (e.g., walking trail, park, fitness club) for engaging in physical activity," "My neighborhood provides a safe and well-maintained environment (e.g., adequate lighting and sidewalks, light traffic volume) for being physically active," "I have access to enjoyable scenery when engaging in physical activity," and "I frequently observe other people exercising." Items were scored on a four-point Likert scale from 1 (strongly disagree) to 4 (strongly agree). The construct validity (GFI = .990, AGFI = .962, RMSEA = .077) of this scale was confirmed by the respondents.

### Statistical Analyses

The analysis of the data involved assessment of replies from the 1,928 adults who had responded fully about the variables or to all the instruments. Data were analyzed using structural equation modeling (SEM) estimated in AMOS 5.0 by type of physical activity. Structural equation modeling was conducted to determine associations between environmental, psychological, and social factors with physical activity. Based on the proposed relationships in the ecological model, the structural equation model included paths related to environmental factors (exogenous variables), self-efficacy, social support, and pros and cons of physical activity (endogenous variables). Also included were paths from environmental factors to the pros and cons of exercise, self-efficacy, and social support; paths from the pros and cons of exercise to self-efficacy and social support; the path from social support to self-efficacy; and the path from self-efficacy to physical activity. Only significant (*P *< .05) bivariate correlations between these variables were integrated to predict physical activity. Path coefficients and correlations were reported as standardized estimates. The model was assessed using GFI, AGFI, RMSEA, and Akaike information criterion (AIC). GFI and AGFI indices were used to measure how well the model fit the data, on a range from 0 to 1. Values of .90 or greater indicated a good model fit [35]. RMSEA is a measure of the discrepancy between a population-based model and a hypothesized model assessed per degree of freedom. An RMSEA score from .05 to .08 was indicative of an acceptable fit and values lower than .05 indicated a good fit [36]. A lower AIC value for a model indicated a better fit compared with other models [37]. A model was considered to fit the data well when the following criteria were met: GFI >.90, AGFI >.90, RMSEA <.06, and lower AIC value compared with competing models. A *p *value of less than 0.05 was considered statistically significant. All statistical analyses were performed with SPSS 12.0J for Windows [38] and Amos 5.0J for Windows [39], SPSS Inc., Chicago, USA.

## Results

### Participant Characteristics

Table 1 shows the characteristics of the participants. For the overall study participant, the mean age (standard deviation; SD) was 43.6 (13.0) years. The percentage of higher educated individuals was 47.7%. The percentage of those who were married was 67.8%, of those living with another person was 87.1%, and of those who were employed was 54.4%. The figure for mean minutes of walking per week (SD) was 222.1 (458.9), for moderate-intensity activity excluding walking per week was 94.3 (333.8), and for vigorous-intensity activity was 55.2 (202.2).

**Table 1:** Descriptive characteristics (numbers and percentages)

	n	%
Overall	1932	100.0
Gender		
Males	962	49.8
Females	970	50.2
Age, group		
20-29	385	20.0
30-39	387	20.1
40-49	393	20.4
50-79	763	39.6
Mean ± SD	43.6 ± 13.0	
Marital status		
Unmarried	621	32.2
Married	1307	67.8
Living condition		
Living with others	1679	87.1
Living alone	249	12.9
Educational level		
4-years university or greater	920	47.7
2-years university or	483	25.1
High school or junior high	525	27.2
Employment status		
Employed	1048	54.4
Not employed	880	45.6
Household income level		
<3,000,000 yen	308	16.0
<5,000,000 yen	546	28.3
<7,000,000 yen	396	20.5
<10,000,000 yen	416	21.6
≥10,000,000 yen	262	13.6
Walking, min/week		
Mean ± SD	222.1 ± 458.9	
Moderate-intensity activity, min/week		
Mean ± SD	94.3 ± 333.8	
Vigorous-intensity activity, min/week		
Mean ± SD	55.2 ± 202.2	

### Structural equation modeling

#### Walking

**Figure 1:**
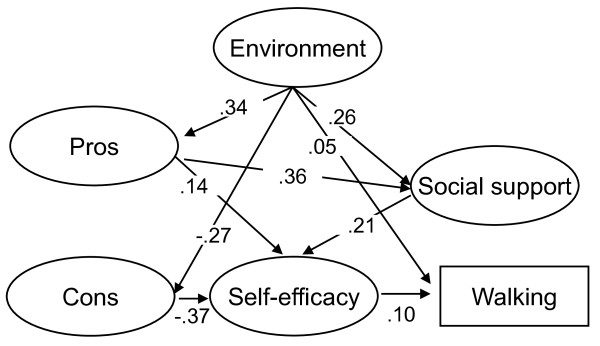
**Environmental, social, and psychological affects on walking**. GFI = .921, AGFI = .908, RMSEA = .047. Only statistically significant paths are indicated in this figure. All paths are statistically significant at p < .05.

Figure 1 shows the results of environmental, social, and psychological influences on walking. All path coefficients are standard partial regression coefficients. With the standard partial regression coefficients, the magnitude of each factor can be directly compared with other factors in the model.

The present study identified no significant associations between environmental factors and self-efficacy or between cons and social support, and no significant association of pros, cons and social support with walking (GFI = .921, AGFI = .908, RMSEA = .047). Recalculation of the model using modified indices reduced the AIC value from 2672.909 to 2671.139. Thus, the final model demonstrated an acceptable fit (GFI = .921, AGFI = .908, RMSEA = .047).

Environmental factors (.051) and self-efficacy were seen to directly affect walking (.104). Cons (-.369) were most effective on self-efficacy, followed by social support (.209) and pros (.143). The path coefficient for the indirect effects of environmental factors on walking through pros and self-efficacy was .005. Through pros, social support, and self-efficacy was .003; through cons and self-efficacy was .010; and through social support and self-efficacy was .006. The total effect of environmental factors on walking was .075.

#### Moderate-intensity activity excluding walking

**Figure 2:**
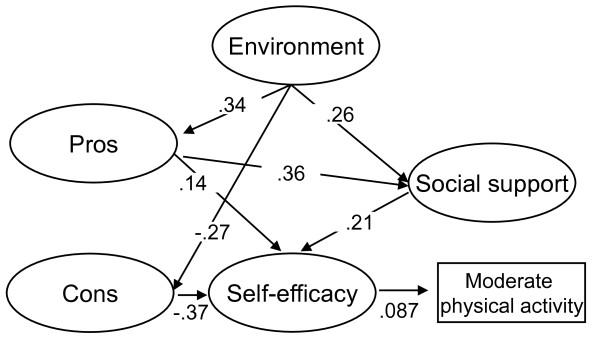
**Environmental, social, and psychological affects on moderateintensity activity excluding walking**. GFI = .921, AGFI = .908, RMSEA = .047. Only statistically significant paths are indicated in this figure. All paths are statistically significant at p < .05.

The moderate-intensity activity excluding walking model was similar to the walking model (figure 2). The present study identified no significant associations between environmental factors and self-efficacy, between cons and social support, and no association of environmental factors, pros, cons, and social support with moderate-intensity physical activity excluding walking (GFI = .921, AGFI = .908, RMSEA = .047). Recalculation of the model using modified indices reduced the AIC value from 2681.579 to 2679.833. Thus, the final model demonstrated an acceptable fit (GFI = .921, AGFI = .908, RMSEA = .047). Environmental factors did not directly affect moderate-intensity activity excluding walking; however self-efficacy directly affected moderate-intensity activity excluding walking (.087). The path coefficient for indirect effects of environmental factor on moderate-intensity activity excluding walking through pros and self-efficacy was .004; through pros, social support, and self-efficacy was .002; through cons and self-efficacy was .009; and through social support and self-efficacy was .003. The total effect of environmental factors on moderate-intensity activity excluding walking was .020.

#### Vigorous-intensity activity

**Figure 3:**
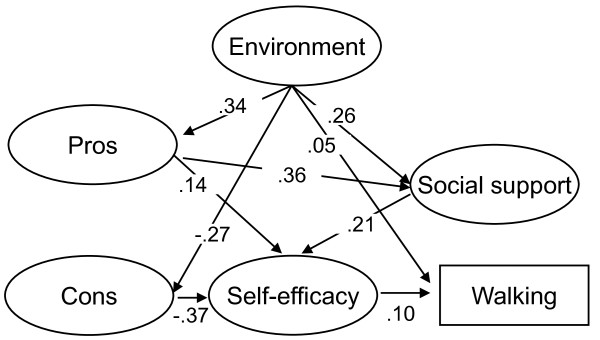
**Environmental, social, and psychological affects on vigorous-intensity activity**. GFI = .921, AGFI = .908, RMSEA = .047. Only statistically significant paths are indicated in this figure. All paths are statistically significant at p < .05.

The vigorous-intensity activity model was similar to the walking and moderate-intensity activity excluding walking model (figure 3). The present study identified no significant associations between environmental factors and self-efficacy; between cons and social support; and no association of environmental factors, pros, cons, and social support with vigorous-intensity physical activity (GFI = .921, AGFI = .908, RMSEA = .047). Recalculation of the model using modified indices reduced the AIC value from 2679.879 to 2675.272. Thus, the final model demonstrated an acceptable fit (GFI = .921, AGFI = .908, RMSEA = .047). Environmental factors did not directly affect vigorous-intensity activity; however self-efficacy directly affected vigorous-intensity activity (.222). The path coefficient for the indirect effects of environmental factors on vigorous-intensity activity through pros and self-efficacy was .011; through pros, social support and self-efficacy was .006; through cons and self-efficacy was .022; and through social support and self-efficacy was .012. The total effect of environmental factors on vigorous-intensity activity was .051.

## Discussion

The present study indicated that environmental factors had indirect effects on walking, moderate-intensity activity excluding walking, and vigorous-intensity activity through self-efficacy for exercise, social support for exercise and pros and cons for exercise among Japanese adults. A previous study [13] suggested that environmental factors indirectly affected walking, moderate-intensity activity, and vigorous-intensity activity through motivation and self-efficacy. Moreover, the availability of physical activity facilities directly affected walking and moderate-intensity activity; also, the quality of a neighborhood directly affected moderate physical activity and vigorous-intensity activity [13]. Research results on direct and mediated effects of perceived equipment accessibility and neighborhood safety with regard to physical activity among adolescent girls indicated that these environmental factors were mediated by the effects of self-efficacy for overcoming barriers [14-16]. The present study investigated the environmental factors that affected moderate to vigorous-intensity activity in males and females aged from 20 to 79 years. While previous studies [14-16] had focused on different levels of intensity of physical activity and age groups, the present study supported the previous finding that environmental factors affect physical activity through psychological factors. The present study found that self-efficacy was the most influential factor that directly affected physical activity. This was consistent with a previous study, thus supporting the hypothesis that self-efficacy plays an important role as a moderate variable of behavior in social cognitive theory [40]. In addition, social support influenced physical activity through self-efficacy. This again replicated the reported finding that social support affects physical activity through self-efficacy and motivation [13,15,41,42].

Findings from the present study indicate that environmental factors directly affect the pros and cons for exercise. Pros for exercise directly affect self-efficacy and social support, and cons for exercise directly affect self-efficacy only. Previous studies had reported a direct relationship between pros and cons and engaging in physical activity [43]. However, there have been no reports on how self-efficacy or social support affects the effects of pros and cons on physical activity. It also remains unclear how pros and cons affect the relationship between physical activity and environmental factors. The findings of the present study suggest that through self-efficacy and social support, pros and cons affect the impact of environmental factors on physical activity.

With regard to the indirect effect of environmental factors on physical activity, the path coefficient was highest from cons to self-efficacy. Perception of environmental factors affected low cons, which in turn affected high self-efficacy and promoted physical activity. This finding suggests that some environmental interventions could reduce cons associated with exercise and thereby promote physical activity in Japanese adults. For instance, these include maintaining neighborhood exercise facilities, improving access to available facilities, promoting better awareness of the facilities, and improving neighborhood safety.

The present study found differences in the model based on the types of physical activities. While a direct and positive effect from environmental factors was found with regard to walking, no such effect was found from vigorous and moderate-intensity activity excluding walking. Further research examining environmental factors associated with physical activity needs to consider these differences based on the types of physical activities when promoting physical activity. For instance, environmental factors conducive to walking can be seen to directly promote walking. On the other hand, for increasing vigorous-intensity activity, environmental factors should be able to reduce cons and increase pros and social support in order to gain high self-efficacy, which directly affects physical activity. Further study is needed to develop and evaluate the effects of specific interventions directed at the environmental factors that promote physical activity.

A few studies [13-16,23-26] have examined the direct and indirect effects of built-environmental, psychological, and social factors on physical activity. Furthermore, no such study has been conducted in Japan, which has important cultural differences from other countries. For example, compared with individuals in Western countries, the prevalence of overweight participants (BMI ≥ 25.0 kg/m^2^) who are more likely to be more inactive people is low; 29.6% in males and 19.0% in females in Japan [2]. Therefore, the present study contributes to the development of physical activity promotion strategies in Japan.

The present study also has a few limitations. First, the present study was conducted in an Internet setting. A potential limitation of Internet surveys is that respondents tend to be young, educated, and with a higher income [44]. The participants in the study may have included a higher proportion of not employed females than those in the general Japanese population. Furthermore, previous studies [45,46] have indicated that participants with high levels of leisure-time sedentary behavior, e.g., Internet and computer use, were often those associated with low levels of physical activity. The adjusted prevalence for age bracket of the Japanese population (according to the 2005 Census data [47]) indicated that 65.0% of the population engaged in ≥ 150 minutes per week of at least moderate-intensity activity.

In the present study, the percentage of individuals who engaged in physical activity was lower: 7.5%. Therefore, the present study participant may have underestimated the affects of environmental, psychological, and social factors on physical activity in comparison with the general Japanese population. Second, the present study used a self-administered questionnaire to examine physical activity; however, the reliability and validity of the scale was comprehensively examined and confirmed in a previous study [30]. Therefore, the possibility of selection bias cannot be excluded. Moreover, in the present study, environmental factors were unable to confirm the existence of the path from moderate to vigorous-intensity activity. This could be because items for environmental factors may have been more heavily associated with walking than with moderate and vigorous-intensity activity. However, this scale for environmental factors included items such as the presence of facilities (walking trails, fitness clubs, etc.) for engaging in physical activity, as well as the availability of home fitness equipment (shoes, pedometers, dumbbells, etc.), which represent environmental factors that affect vigorous-intensity activity. Therefore, content validity appears to be appropriately retained. In addition, although outcome variables assessed items with reference to the physical activity, the psychological and social variables assessed items with reference to exercise in the present study. This may cause weak associations between these variables and physical activity. In future research, the outcome variables and the psychological and social variables should be matched.

Respondents of the present study were considered as a representative section of the population because the Internet research service organization that conducted the present study used randomly selected individuals from a pool of 1,150,000 so that an equal number of responses were obtained from each gender and age group between 20 to 79 years. Moreover, the respondents included those living in various regions with diverse occupations, and 71.6%, 76.6%, 75.9%, 69.5%, and 57.7% of the Japanese population in their 20's, 30's, 40's, 50's, and 60's-80's, respectively, used the Internet at least once per week [48]; this further strengthens the representativeness of the survey. Since no other study of the Japanese population has been conducted on this topic, the findings of the present study will be important for the development of intervention strategies for population-based health promotion in the future and will therefore contribute to the promotion of physical activity.

## Conclusions

The present study indicates that environmental factors could be shown to have indirect effects on walking, moderate-intensity activity excluding walking and vigorous-intensity activity through self-efficacy for exercise, social support for exercise and pros and cons for exercise among Japanese adults. Environmental factors had indirect effects on physical activity through self-efficacy, social support, and pros and cons. The strongest indirect effects could be seen by examining the paths of environmental factors on physical activity through cons to self-efficacy. The total effects of environmental factors on physical activity were 0.02 on walking, 0.02 on moderate-intensity activity excluding walking and 0.05 on vigorous-intensity activity. The findings of the present study imply that intervention strategies to promote more engagement in physical activity for population-based health promotion may be necessary.

## Competing interests

The authors declare that they have no competing interests.

## Authors' contributions

KI participated in the design of the study, performed the statistical analyses, and drafted the manuscript. AS and KO conceived the study, participated in its design and coordination, and helped in drafting the manuscript. All the authors have read and approved the final manuscript.
